# Lipopolysaccharide induces bacterial autophagy in epithelial keratinocytes of the gingival sulcus

**DOI:** 10.1186/s12860-018-0168-x

**Published:** 2018-08-30

**Authors:** Kanako Hagio-Izaki, Madoka Yasunaga, Masahiro Yamaguchi, Hiroshi Kajiya, Hiromitsu Morita, Masahiro Yoneda, Takao Hirofuji, Jun Ohno

**Affiliations:** 1 Section of General Dentistry, Department of General Dentistry, Fukuoka Dental College, Fukuoka, Japan; 2 Research Center for Regenerative Medicine, Fukuoka Dental College, Fukuoka, Japan; 3 Section of Orthodontics, Department of Oral Growth and Development, Fukuoka Dental College, Fukuoka, Japan; 4 Section of Geriatric Dentistry, Department of General Dentistry, Fukuoka Dental College, Fukuoka, Japan; 5 Section of Cellular Physiology, Department of Physiological Science and Molecular Biology, Fukuoka Dental College, Fukuoka, Japan

**Keywords:** Gingival sulcus, Keratinocytes, Lipopolysaccharide, Autophagy, Toll-like receptors (TLRs), AMP-activated protein kinase (AMPK), Reactive oxygen species (ROS), *Porphyromonas gingivalis*

## Abstract

**Background:**

Interactions of resident bacteria and/or their producing lipopolysaccharide (LPS) with sulcular epithelial keratinocytes may be regulated by autophagy in the gingival sulcus. In this study, we investigated an induction of bacterial autophagy in exfoliative sulcular keratinocytes of the gingival sulcus and cultured keratinocytes treated with *Porphyromonas gingivalis*-originated LPS (PgLPS).

**Results:**

Exfoliative sulcular keratinocytes showed an induction of autophagy, in addition to increased expression of LPS-mediated factors including lipopolysaccharide-binding protein and toll-like receptors (TLRs), leading to co-localization of bacteria with autophagosomes. In contrast, exfoliative keratinocytes from the free gingiva did not show similar autophagy. Autophagy activity in human cultured keratinocyte cells (HaCaT) was induced by PgLPS, which was dependent partially on the AMP-activated protein kinase (AMPK) pathway via increased intracellular reactive oxygen species (ROS) and was in association with an activation of TLR4 signaling. After incubation of cultured keratinocytes with *E.coli* BioParticles following PgLPS stimulation, co-localization of bioparticles with autophagosomes was enhanced. Conversely, blockage of autophagy with 3-methyladenin and LPS-binding with polymyxin B led to significant reduction of co-localization of particles with autophagosomes.

**Conclusion:**

These findings indicate that PgLPS-induced autophagy is at least partially responsible for interaction between bacteria and sulcular keratinocytes in the gingival sulcus.

## Background

Autophagy is an intracellular degradation process by which cytosolic materials including damaged organelles, toxic protein aggregates, and intracellular bacterial or viral pathogens are sequestered in a specialized double-membrane-bound autophagosomes and delivered to lysosomal compartments [1–3]. A growing body of evidence has revealed that autophagy plays an important role in cellular processes such as aging, development, inflammation, and innate immunity [4–6]. Some studies have suggested that autophagy also contributes to a range of disease states including microbial infection [7, 8]. The recently identified process of autophagy involves recognition of specific cargo such as invasive, intracellular bacteria by the autophagosome/lysosome pathway for degradation (‘xenophagy’), protecting the host against pathogen colonization [9, 10]. Bacterial autophagy has been highlighted as a fundamental host cell response to bacterial invasion including that by *Mycobacterium tuberculosis* [11], *Salmonella enterica* [4], Group A *Streptococcus* [5], or *Listeria monocytogenes* [12].

Periodontitis is the most prevalent inflammatory condition among oral diseases, affecting 30 to 40% of the population over 35 years of age, and is typically characterized by breakdown of tooth-supporting tissues, resulting in a loss of dentition [13]. Gram-negative anaerobic bacteria such as *Bacteroides forsythus* and *Porphyromonas gingivalis* (*P. gingivalis*) are well-established periodontal pathogens, and great numbers of these bacteria are found in dental plaque in the gingival sulcus [14]. In gingival sulcus, an interaction between periodontal bacteria and sulcular epithelium is the first step during development of periodontitis. The sulcular epithelium consists of a stratified squamous epithelium and is neither keratinized nor terminally differentiated, unlike other oral epithelial keratinocytes [15]. The sulcular epithelium is affected by accumulation of bacteria in the gingival sulcus. Direct interaction of periodontal bacteria with the sulcular epithelium leads to adhesion and invasion of bacteria into sulcular epithelial keratinocytes.

Lipopolysaccharide (LPS) is the biologically active constituent of endotoxins derived from the cell wall of Gram-negative bacteria and plays an important role in pathogenesis of various infections [16, 17]. Among numerous bacteria in the gingival sulcus, *P. gingivalis* generates large amounts of LPS (*P. gingivalis-*originated LPS, PgLPS) in the outer membrane. Although the biological potential of PgLPS is lower than that of LPS from *Escherichia coli*, PgLPS leads to development of periodontitis and induction of several immunological events. It is known that LPS is a potent inducer of autophagy in several cell lines including macrophages [16], human keratinocytes [18], and myoblasts [19]. Recent studies have discussed a beneficial role of autophagy in gingival fibroblasts during development of periodontal complications [20, 21]. However, involvement of autophagy in interactions between bacteria and sulcular keratinocytes has not been studied yet.

This study had two main goals. First, to examine whether interaction between bacteria and sulcular epithelium is observed in the gingival sulcus, we used exfoliative cells from the gingival sulcus and the free gingiva to access expression of LPS-mediated factors and co-localization of bacteria with autophagosomes. Second, we investigated whether autophagy was accelerated in cultured keratinocytes treated with PgLPS leading to recruitment of bacterial particles into autophagosomes.

## Methods

### Reagents and antibodies

High-glucose Dulbecco’s modified Eagle’s medium (DMEM) and *P. gingivalis*-originated lipopolysaccharide (PgLPS) were purchased from Wako Chemicals (Osaka, Japan). Fetal bovine serum (FBS) was purchased from HyClone Laboratories, Inc. (South Logan, UT, USA). 3-methyladenine (3-MA), N-acetylcysteine (NAC), polymyxin B sulfate salt (PMB), Hoechst 33324, 4′, 6-diamidine-2′-phenylindole dihydrochloride (DAPI), and monoclonal antibody against β-actin (ACTB) were purchased from Sigma-Aldrich (St. Louis, MO, USA). Mouse anti-human lipopolysaccharide-binding protein (LBP) monoclonal antibody was purchased from Biometec (Greifswald, Germany). Mouse anti-human toll-like receptor 2 and 4 (TLR-2 and TLR-4) monoclonal antibodies were obtained from Abcam (Cambridge, UK). Trypan blue solution (0.4%) and protein assay kit were purchased from Pierce (Hercules, CA, USA), and pHrodo™*E.coli*BioParticles™Conjugate for phagocytosis was purchased from ThermoFisher Scientific (Waltham, MA, USA). Rabbit polyclonal antibodies against microtubule-associated protein light chain 3 (LC3) and beclin-1 (BECN-1) were purchased from MBL (Tokyo, Japan). Precision Plus Protein Western C Standard, 4–20 and 12% Mini-protean TGX gels, horseradish peroxidase-conjugated anti-mouse and -rabbit secondary antibodies, and Trans-Blot Transfer Packs were obtained from Bio-Rad (Richmond, CA, USA). Cell Lysis Buffer, 1× protease/phosphatase inhibitor cocktail, Signal Fire Plus ECL Reagent, rabbit anti-AMP-activated protein kinase α (AMPK) polyclonal antibody, and rabbit anti-phospho AMPα (Thr172; pAMPK) monoclonal antibody were purchased from Cell Signaling Technology (Delaware, CA, USA). Mouse anti-human cytokeratin 14 (CK14) and intercellular adhesion molecule-1 (ICAM-1) were obtained from Agilent (Santa Clara, CA, USA). Alexa Flour 488 or 568-conjugated goat anti-rabbit IgG and rabbit anti-mouse IgG and CellROX®GreenReagent were purchased from Invitrogen (Carlsbad, CA, USA).

### Ethics statement

The study was approved by the Ethics Review Board of Fukuoka Dental College (No. 298). All studies involving human participants were conducted in full compliance with the Declaration of Helsinki. All participants completed an informed consent form.

### Collection of exfoliative cells from human gingiva

Oral smears were taken from two gingival sites (gingival sulcus and free gingiva) using the EndoCervix-Brush (Rover Medical Devices, KV Oss, The Netherlands) in 30 volunteers (15 males and 15 females) from Fukuoka Dental College. In all cases the gingival mucosa appeared periodontitis-free and clinically normal. Liquid based cytology (LBC) was used on oral smears collected by the cytobrushes. Exfoliated brushes were immersed and washed in CytoRich Red Fluid to scatter cells in the solution. After centrifugation, cell pellets were suspended in distilled water. Cell suspensions were placed on slide glass and re-fixed in 95% ethanol. Papanicolaou (Pap) and May-Gieamsa stains were used for cytopathology.

### Cell culture

HaCaT cells, a human keratinocyte line, were maintained in DMEM with 10% (*v*/v) FBS and 1% penicillin and streptomycin (Anti-Anti) at 37 °C in a humidified incubator with 5% CO_2_. For PgLPS stimulation, HaCaT cells at approximately 80% confluence were exposed to PgLPS at different concentration (0, 0.5, 1.0, 5.0, 10, or 50 μg/ml) for 24 h.

### Cell viability assay

To determine cell viability, HaCaT cells treated with or without PgLPS were washed with phosphate-buffered saline (PBS), harvested from the dishes via trypsinization, resuspended in PBS, and diluted 1:1 in 0.4% trypan blue solution. Cell viability was calculated using a Countess®Automated Cell Counter (Invitrogen) following the manufacturer’s instructions.

### Immunocytochemistry of exfoliative and cultured cells

For immunocytochemical analysis of exfoliative cells, exfoliative brushes were immersed and washed in PBS. Cell suspensions of both exfoliative and cultured HaCaT cells were placed on glass slides and fixed in cold acetone. Both cells were incubated with primary antibodies (LBP, ICAM-1, TLR4, TLR2, and LC3, 1:100) at 4 °C overnight. After washing with PBS, the cells were incubated with anti-rabbit IgG or anti-mouse IgG conjugated with Alexa Fluor 488 or 568 at room temperature for 45 min. To visualize nuclei, cells were counterstained with Hoechst 33342.

### Immunofluorescent detection for co-localization of Bacteria and autophagosomes

To investigate co-localization of bacteria and autophagosomes, double staining using LC3 antibody and 4,6-diamidino-2-phenylindole (DAPI) was performed in exfoliative cells. Exfoliative cells fixed on slides were first incubated with anti-LC3 antibody for 6 h at room temperature. After washing with PBS, the cells were incubated with a mixture of anti-rabbit IgG conjugated with Alexa Fluor 488 (1:200) and DAPI (5 μg/ml) for 45 min at room temperature.

### Western blot analysis

Both exfoliative and cultured cells were lysed in Cell Lysis Buffer containing protease and phosphatase inhibitor (1× Protease/Phosphatase Inhibitor Cocktail). Protein concentration was measured with a protein assay kit. Equal amounts (15 μg) of protein along with a protein marker (Precision Plus Protein Western C Standards) were separated on Mini-protean TGX gels for 30 min at 200 V. The separated proteins were transferred to a polyvinylidene fluoride (PVDF) membrane using the Trans-Blot Turbo Transfer system (Bio-Rad) with Trans-Blot Transfer Packs. Western blots were processed on the iBind Western System (Life Technologies, Carlsbad, CA, USA) with primary antibodies and horseradish peroxidase-conjugated secondary antibodies. The protein bands were developed using an enhanced chemiluminescence system (SignalFire Plus ECL Reagent). Band density was quantified using the software NIH-Image J.

### Analysis of intracellular reactive oxygen species (ROS) production

Fluorescent assay of intracellular ROS production was performed using a CellROX®GreenReagent following the manufacturer’s manual. Production of intracellular ROS was detected as green-stained cells in fluorescence microscopy.

### Immunofluorescent detection of bacterial co-localization with autophagosomes

To demonstrate that PgLPS-induced autophagy can promote increased co-localization of bacteria with autophagosomes, cultured keratinocytes treated with or without PgLPS were infected with *E. coli* (K-12 strain) BioParticles at a MOI of 20:1 for 1 h. Subsequently, the cells were washed with PBS and were immunocytochemically stained with anti-LC3 antibody, followed by incubation with Alexa Flour 488 conjugated anti-rabbit IgG. Co-localization was confirmed using fluorescence microscopy.

### Statistical analysis

Statistical analysis was done using the software STATVIEW (STATVIEW for Windows, version 5). The analysis was performed using two-way analysis of variance (ANOVA) and Scheffe’s multiple comparesion test or Student’s t-test to determine the statistical differences among samples. Data were represented as mean ± standard deviation (SD) and *P*-values < 0.05 were considered to be statistically significant.

## Results

### Exfoliative keratinocytes in the gingival sulcus

**Fig. 1:**
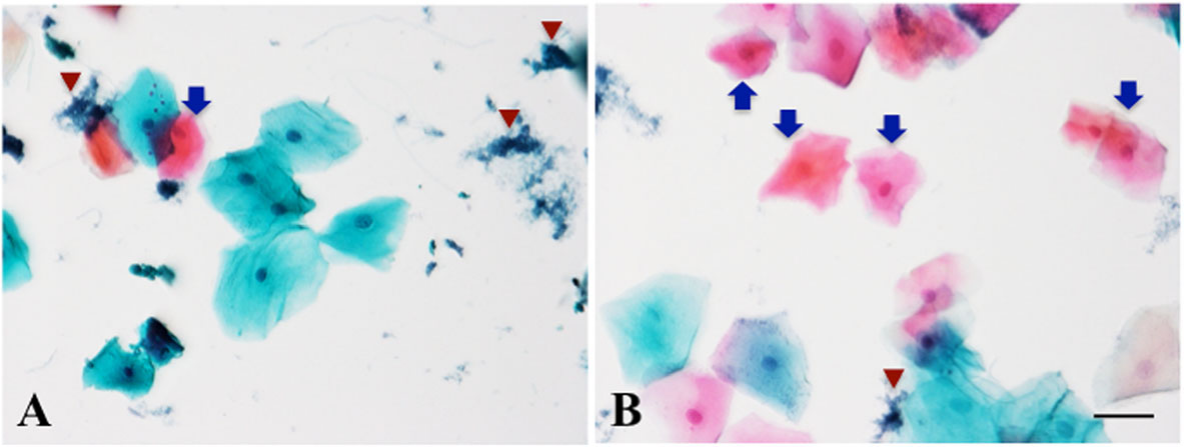
Exfoliative cells in the human gingiva. Representative images of exfoliative cells, stained with Pap staining, from gingival sulcus (**a**) and free gingiva (**b**). Blue arrows, keratinized cells; red arrow heads, bacterial colonies. Bar = 50 μm

The gingival sulcus is the natural space formed between the tooth and the surrounding gingival tissue and is a landing spot for plaque containing numerous colonies of bacteria. Surfaced sulcular epithelium must always be directly stimulated by bacteria and/or bacterial products. We first examined exfoliative cytopathology in the gingival sulcus and free gingiva, which is a portion of gingiva surrounding the tooth but is not directly attached to the tooth surface. The surfaced sulcular epithelium consisted of superficial squamous cells containing very few keratinized cells (Fig. 1a), while epithelial cells from the free gingiva contained several keratinized cells (Fig. 1b). The sulcular specimens showed a large number of bacterial colonies (Fig. 1a) compared with free gingival specimens (Fig. 1a and b).

### Exfoliative sulcular keratinocytes interact with resident bacteria in the gingival sulcus

**Fig. 2:**
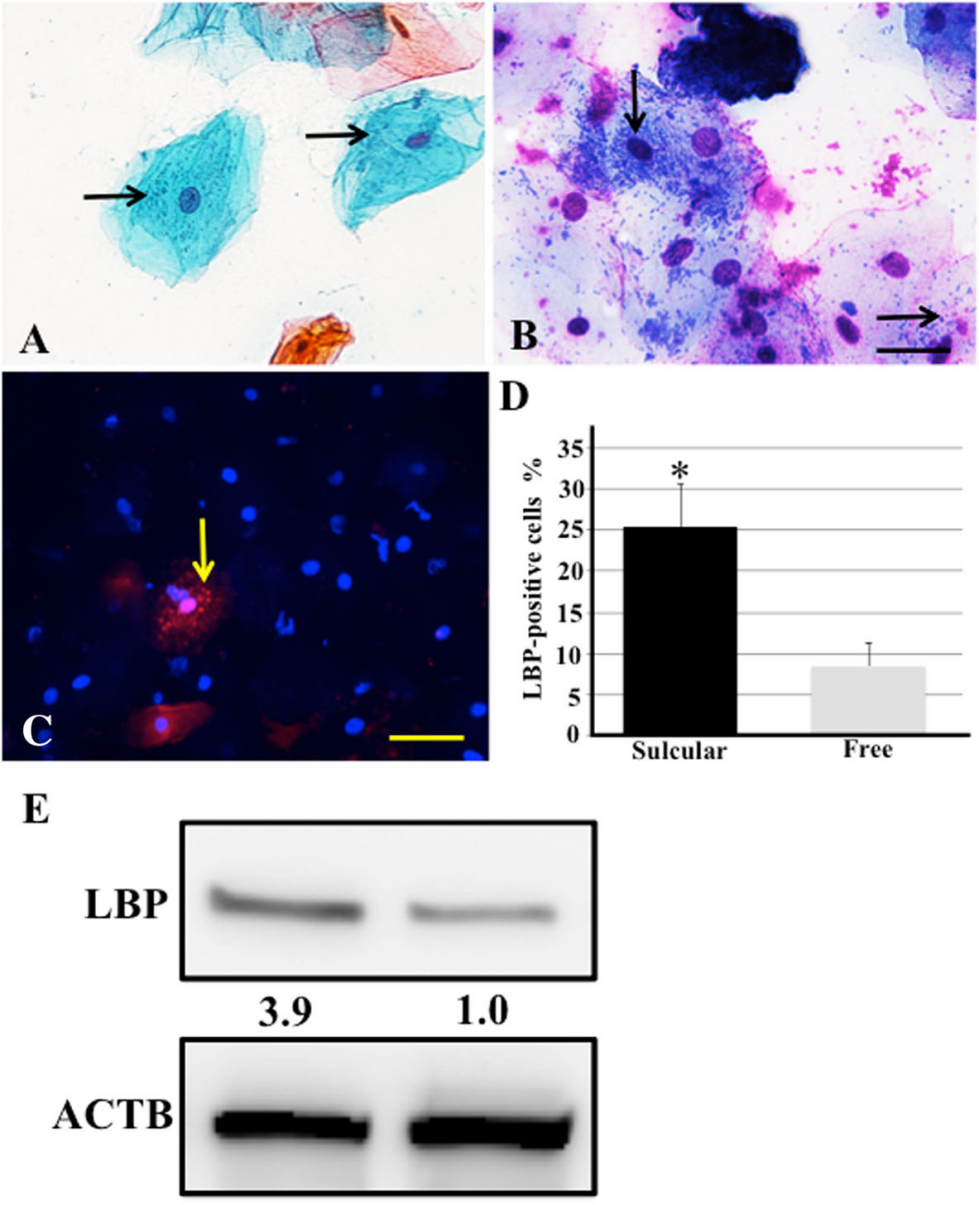
Intracellular detection of bacterial pathogens in exfoliative sulcular keratinocytes. **a** and **b** Representative images of exfoliative epithelial keratinocytes stained with Pap (**a**) and May-Giemsa (**b**) staining. Arrows, intracytoplasmic bacterial particles. Bar = 50 μm. **c** Immunocytochemical evaluation of LBP expression in the exfoliative sulcular keratinocytes. LBP antibody staining showed red-colored positive particles in the cytoplasm of sulcular cells. The nucleus was stained with Hoechst 33342 (blue). Arrows, intracytoplasmic bacterial particles. Bar = 50 μm. **d** Quantitative analysis of percentage of LBP-positive cells in exfoliative sulcular keratinocytes. The mean number of percentage of LBP-positive cells ± SD from five independent studies. *Significantly different at *P* < 0.05 compared with exfoliative cells from the free gingiva. **e** Western blotting analysis of LBP expression in exfoliative keratinocytes from gingival sulcus and free gingiva. β-actin (ACTB) was used as a loading control. Band densities were presented as LBP expression fold-increases (normalized to ACTB) and compared with the results for exfoliative cells from free gingiva. Quantification results are shown below the corresponding blots. All experiments were performed in quadruplicate

Some of exfoliative keratinocytes in the gingival sulcus showed intracellular bacterial particles with Pap staining (Fig. 2a). With May-Giemsa staining, which is known to be useful in detecting bacteria, Giemsa-stained fine, dark blue particles were observed in some sulcular keratinocytes (Fig. 2b). These findings indicated that sulcular keratinocytes positively interact with bacteria residing in the gingival sulcus. We performed immunocytochemical analysis of LPS-binding protein (LBP) in the exfoliative sulcular keratinocytes to examine whether the intracellular bacterial particles had originated from bacterial pathogens. LPS is one of the predominant products generated from resident bacteria in the gingival sulcus. Intracytoplasmic particles, shown in Fig. 2a and b, were reactive immunocytochemically with anti-LBP antibody in exfoliative sulcular keratinocytes (Fig. 2c). As shown in Fig. 2d, percentage of LBP-positive cells was increased in exfoliative sulcular keratinocytes, compared with cells from free gingiva (25.4 ± 5.2% [sulcular cells] versus 8.4 ± 2.9% [cells from free gingiva], *P* = 0.032). In Western blotting analysis, increased LBP expression was observed in the exfoliative sulcular keratinocytes, compared with keratinocytes from the free gingiva (Fig. 2e). These findings suggested that interaction between sulcular keratinocytes and resident bacteria results in internalization of bacterial pathogens into those cells.

### LPS-induced factors were expressed in exfoliative sulcular keratinocytes

**Fig. 3:**
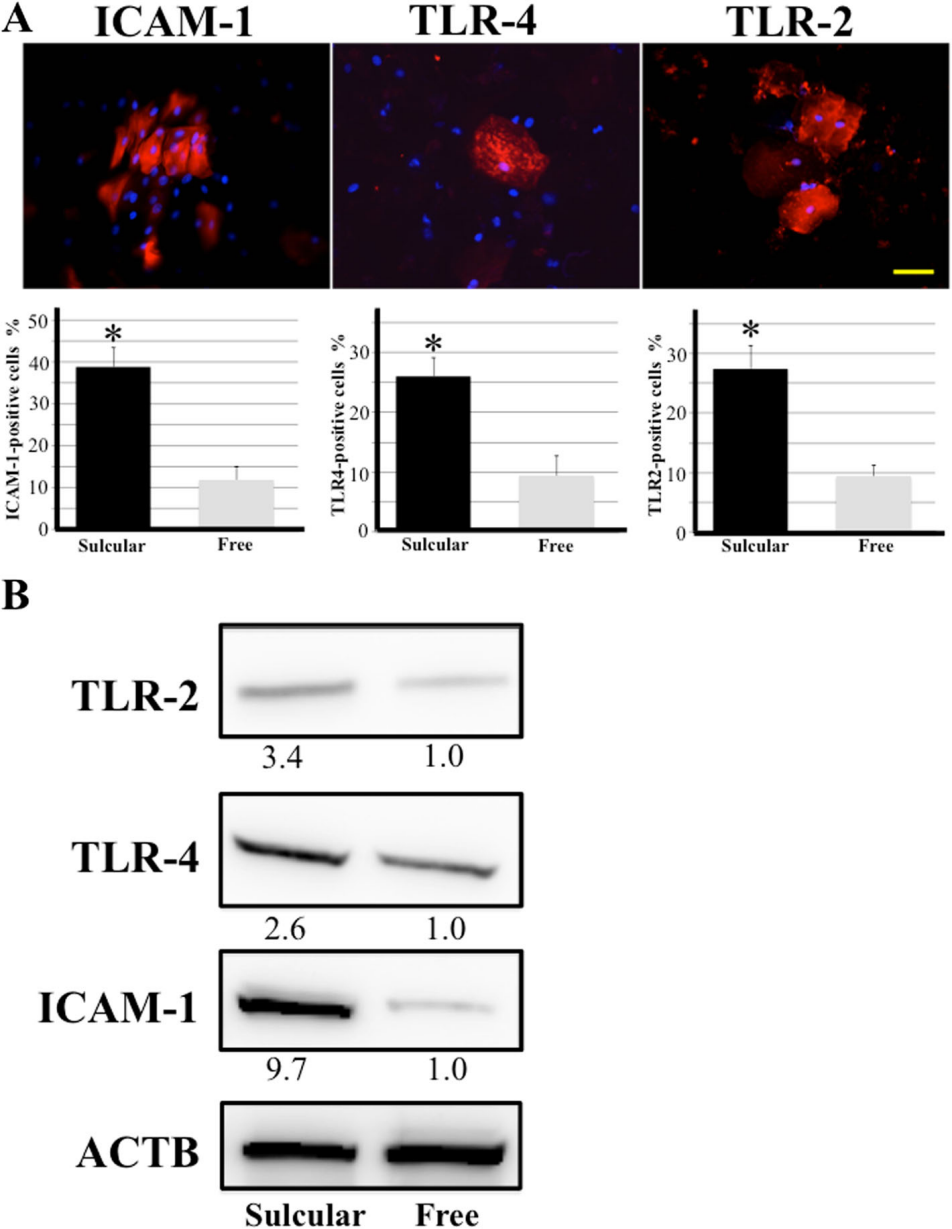
Expression of LPS-induced factors in exfoliative sulcular keratinocytes. **a** Immunocytochemical detection of ICAM-1, TLR-4, and TLR-2 expression (red) in sulcular epithelial cells. The nucleus was stained with Hoechst 33342 (blue). Bar = 50 μm. Graphs show the quantitative analysis of the percentage of positive cells. Mean number of the percentage of each positive cells ± SD from five independent studies. *Significantly different (Student’s t-test) at *P* < 0.05 compared with exfoliative cells from free gingiva. **b** Western blotting analysis of ICAM-1, TRL-4, and TLR-2 expression in exfoliative cells from gingival sulcus and free gingiva. β-actin (ACTB) was used as a loading control. Band densities were presented as antibodies’ expression fold-increases (normalized to ACTB) and compared with the results for exfoliative cells from free gingiva. Quantification results are shown below the corresponding blots. All experiments were performed in quadruplicate

We examined expression of ICAM-1, TRL-4, and TLR-2 in exfoliative sulcular keratinocytes to elucidate the effect of bacterial LPS on the sulcular epithelium. Immunocytochemical assay showed cytoplasmic expression of these factors in the exfoliative sulcular keratinocytes (Fig. 3a). The percentage of positive cells was higher in sulcular cells than in cells from free gingiva (Fig. 3a). Western blotting also showed increased expression of these factors in exfoliative sulcular keratinoctytes compared with that in keratinocytes from free gingiva (Fig. 3b).

### LC3 expression was increased in exfoliative sulcular keratinocytes

**Fig. 4:**
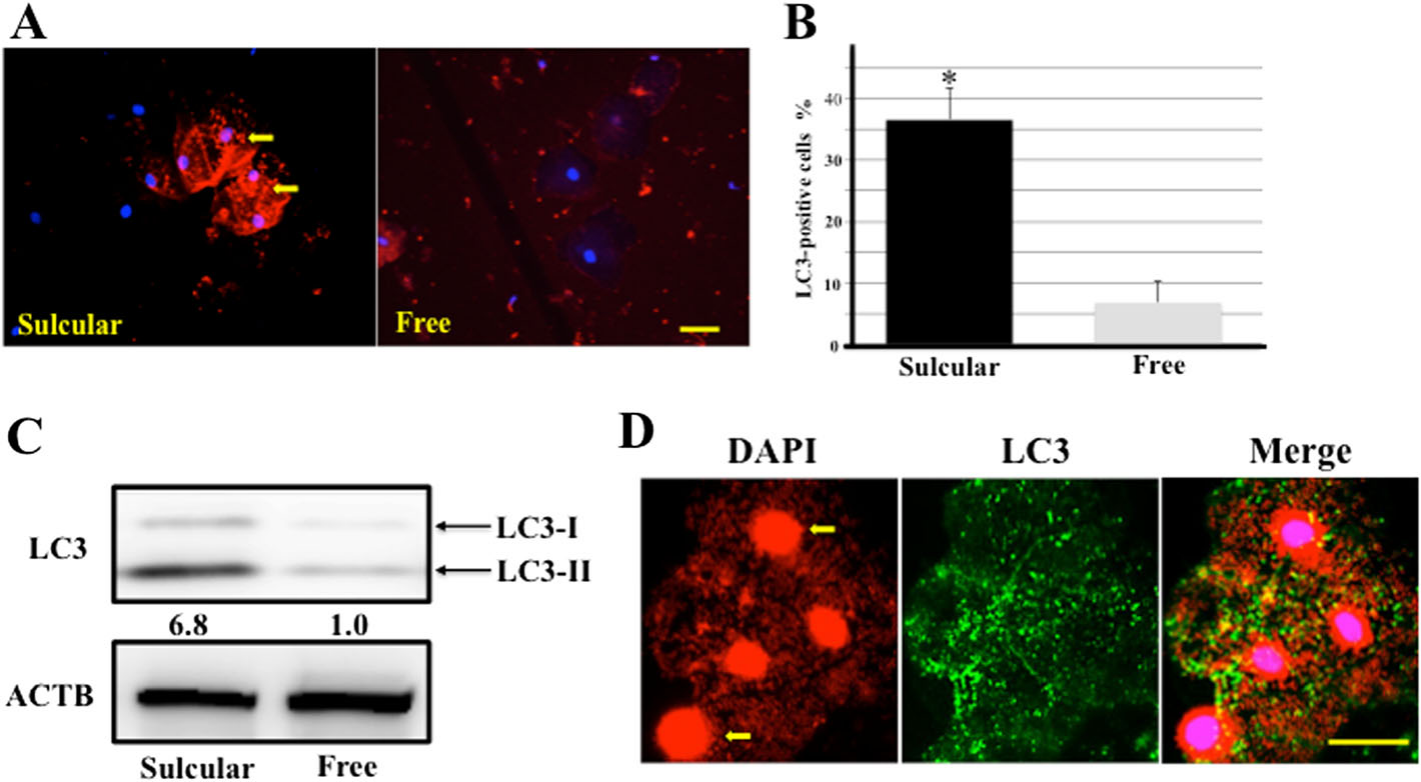
LC3 expression in exfoliative sulcular keratinocytes. **a** Immunofluorescence of LC3 expression (red) in exfoliative cells from gingival sulcus (Sulcular) and free (Free) gingiva. The nucleus was stained with Hoechst 33342 (blue). Arrows, LC3-positive autophagosomes. Bar = 20 μm. **b** Quantitative analysis of percentage of LC3-positive cells in exfoliative cells from gingival sulcus and free gingiva. Mean number of the percentage of LC3-positive cells ± SD from five independent studies. *Significantly different at *P* < 0.05 compared with exfoliative cells from the free gingiva. **c** Western blotting analysis of LC3 expression in exfoliative cells from gingival sulcus and free gingiva. β-actin (ACTB) was used as a loading control. Band densities were presened as LC3 expression fold-increases (normalized to ACTB) and compared with the results for exfoliative cells from free gingiva. Quantification results are shown below the corresponding blots. All experiments were performed in quadruplicate. **d** Fluorescent localization of DAPI-positive bacteria (red) and LC3-positive autophagosomes (green) in exfoliative sulcular cells. Arrows, DAPI-positive nuclei (red) in sulcular keratinocytes. Bar = 20 μm

We next performed an immunocytochemical staining of LC3, one of the reliable markers of autophagosomes, in both exfoliative cells from gingival sulcus and free gingiva to examine whether autophagy was induced in keratinocytes contacted with bacteria in the gingival sulcus. Particle-like materials stained with LC3 were observed in the cytoplasm of exfoliative sulcular keratinocytes, whereas no reactive materials were found in the exfoliative keratinocytes from the free gingiva (Fig. 4a). As shown in Fig. 4b, the percentage of LC3-positive cells was increased in the exfoliative sulcular epithelium compared with epithelium from free gingiva (36.6 ± 5.2% [sulcular cells] versus 6.8 ± 3.3% [cells from free gingiva], *P* = 0.022). In Western blotting analysis, increased LC3 expression was observed in exfoliative sulcular keratinocytes compared with keratinocytes from free gingiva (Fig. 4b). These results suggested that sulcular keratinocytes were exposed to resident bacteria such as LPS-producing bacteria, causing induction of autophagy indicated by formation of autophagosomes in the cytoplasm of those cells.

We next examined whether intracellular bacteria were harbored in autophagosomes in exfoliative sulcular keratinocytes with double staining using LC3 antibody and DAPI staining. DAPI specifically binds to double-stranded DNA, emitting fluorescence when excited by 365-nm UV light, and is used to detect microbial cells in various samples [22]. As shown in Fig. 4d, although DAPI was bound to nuclei in sulcular epithelial cells, DAPI-stained fine particles were observed in the cytoplasm of exfoliative sulcular keratinocytes, indicating that DAPI can bind to bacterial DNA. Merger of DAPI and LC3 double staining showed that numerous DAPI-stained bacteria were resident in LC3-positve autophagosomes. Based on these findings, we suggest that after internalization of bacteria, numerous intracellular bacteria reside in autophagosomes in sulcular keratinocytes.

### PgLPS treatment induced autophagy in cultured human keratinocytes (HaCaT cells)

**Fig. 5:**
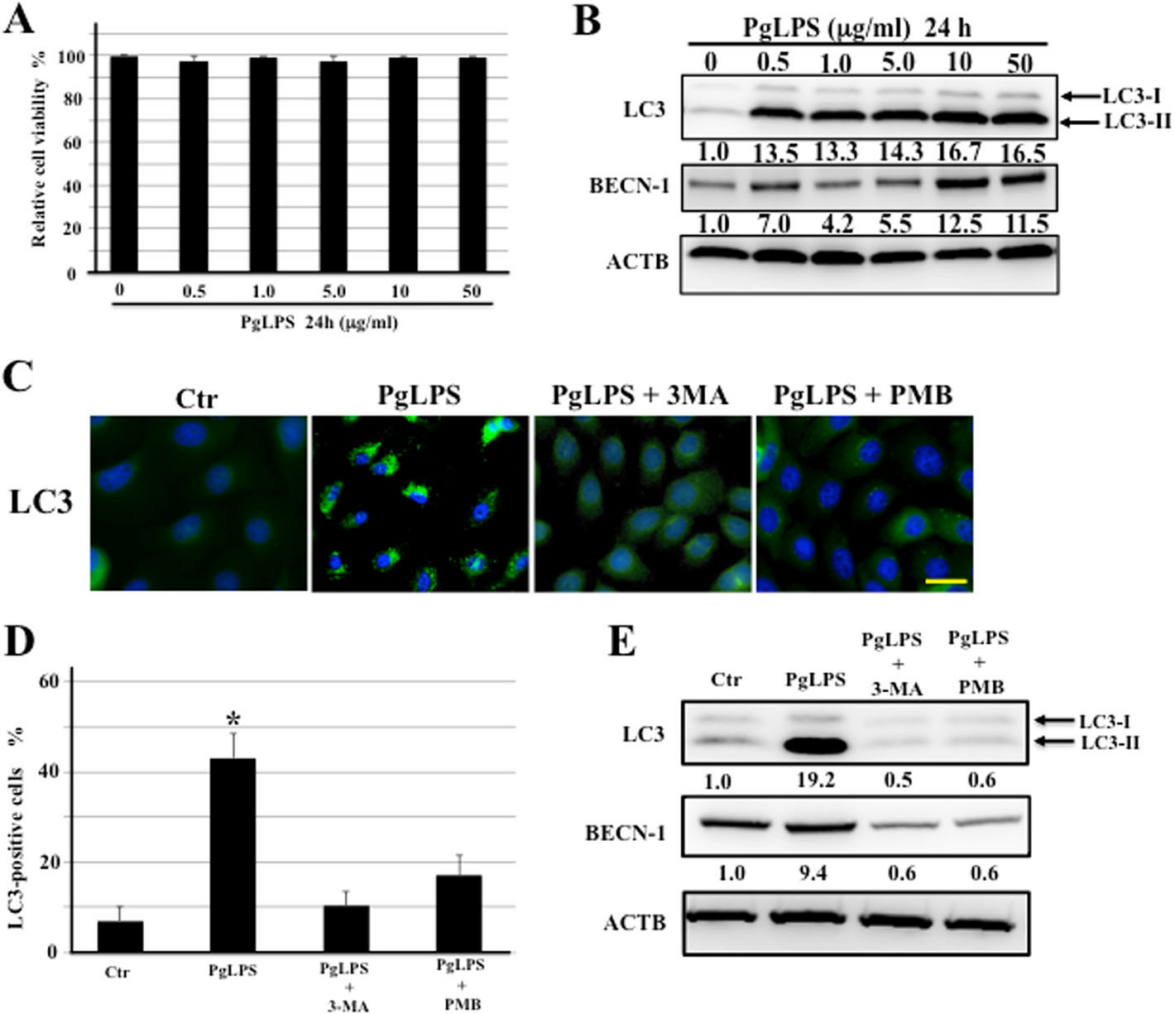
Autophagy induction in cultured keratinocytes by PgLPS. **a** Cell viability was determined using trypan blue exclusion. The graph shows the viability of HaCaT cells exposed different concentrations (0, 0.5, 1.0, 5.0, 10, or 50 μg/ml) of PgLPS for 24 h. Results are presented as a percentage of the trypan blue exclusion in untreated cells. Data represent mean values ± SD in quadruplicate. **b** Western blotting analysis of LC3 and beclin-1 (BECN1) expression in HaCaT cells treated with different concentration of PgLPS for 24 h. β-actin (ACTB) was used as a loading control. Qauntification values shown in part were the fold increases normalized to those of ACTB on day 0. Results are shown below the corresponding blots. All experiments were performed in quadruplicate. **c**-**e** Effect of 3-MA or PMB on induction of autophagy in PgLPS-stimulated HaCaT cells. HaCaT cells were treated with 10 μg/ml PgLPS with or without 10 mM 3-MA or 100 μg/ml PMB for24 h. **c** Immunocytochemical detection of LC3-positive autophagosomes (green) in keratinocytes. Bar = 50 μm. **d** Quantitative analysis of the percentage of LC3-positive cell ± SD from five independent studies. *Significantly different at *P* < 0.05 compared with HaCaT cells treated without PgLPS and treated with 3-MA or PMB. **e** Western blotting analysis of LC3 and BECN-1 expression. β-actin (ACTB) was used as a loading control. Band densities were presened as LC3 or BECN-1 expression fold-increases (normalized to ACTB) and compared with the results for untreated HaCaT cells. Quantification results are shown below the corresponding blots. All experiments were performed in quadruplicate

LPS is known to be a potent inducer of autophagy in several cell lines [10, 16]. To elucidate effects of PgLPS on induction of autophagy in epithelial cells, we cultured the human keratinocyte cell line (HaCaT) because the gingival epithelium including the sulcular epithelium consists of keratinocytes, similar to epidermis of the skin. We first examined effect of PgLPS on cell viability of keratinocytes. Trypan blue dye exclusion showed no significant changes in the cell viability (Fig. 5a). We performed western blotting analysis of LC3 and beclin-1 in keratinocytes. Following treatment of keratinocytes with PgLPS at concentrations of 0, 0.5, 1.0, 5.0, 10, or 50 μg/ml for 24 h, western blotting analysis also showed the concentration-dependent increase in expression of LC3-II and beclin-1 (Fig. 5b). Based on the results of the viability assay and western blotting, a concentration of 10 μg/ml PgLPS was chosen for further experiments.

To elucidate whether PgLPS-induced autophagy can be suppressed by inhibitors of autophagy progression and LPS-binding, we used 3-methyladenine (3-MA) and polymyxin B (PMB), known as inhibitor for autophagy and LPS receptors, respectively. Figure 5c shows autophagosome formation in keratinocytes treated with PgLPS with immunocytochemical detection of LC3. HaCaT cells were incubated with 10 μg/ml PgLPS containing10mM 3-MA or 100 μg/ml PMB for 24 h. 3-MA or PMB treatment remarkably reduced LC3 expression in keratinocytes. The percentage of LC3-positive cells in untreated keratinocytes was 43.2 ± 5.3%, while in keratinocytes pretreated with 3-MA or PMB, LC3-positive percentage was 10.4 ± 3.1% (*P* = 0.026) or 17.4 ± 4.3% (*P* = 0.036), respectively (Fig. 5d). In line with immunocytochemical data, expression of LC3-II and beclin-1 was found to be down regulated by both 3-MA or PMB treatment in western blotting assay (Fig. 5e).

### Upregulation of AMPK activity through ROS accelerated PgLPS-induced autophagy

**Fig. 6:**
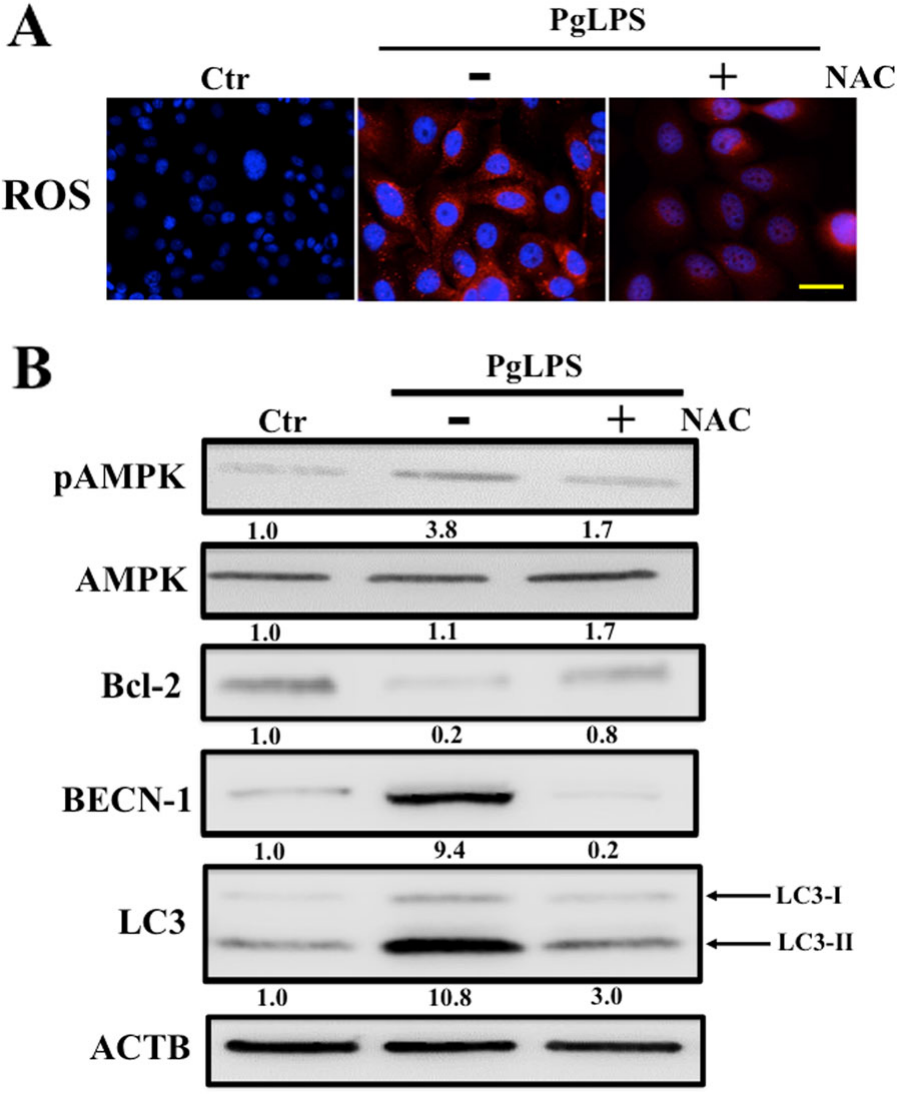
Intracellular reactive oxygen species (ROS) production regulates PgLPS-induced autophagy. HaCaT cells were either treated with 10 μM PgLPS alone or 10 μM PgLPS plus 100 μM N-acetylcystein (NAC) for 24 h. Cells treated with medium alone were control (Ctr). **a** Representative fluorescence images are shown to demonstrate an intracellular ROS production (red) using CellROX reagents. Nuclei were stained with Hoechst 33324 (blue). Bar = 50 μm. **b** Western blotting assay was performed with antibodies against phosphorylated AMPK (p-AMPK), AMPK, Bcl-2, beclin-1 (BECN-1), or LC3. β-actin (ACTB) was used as a loading control. Band densities were presened as antibodies’ expression fold-increases (normalized to ACTB) and compared with the results for untreated cells (Ctr). Quantification results are shown below the corresponding blots. All experiments were performed in quadruplicate

Recent studies have revealed that intracellular ROS production is implicated in mediating induction of autophagy in response to various cellular stresses [23]. First, we examined whether intracellular ROS can be produced in PgLPS-stimulated keratinocytes using the fluorescent assay with CellROX reagents. As shown in Fig. 6a, PgLPS-treated cells showed increased levels of intracellular ROS, whereas ROS production was decreased in cells treated with NAC, known as anti-ROS activity resulting from its free radical scavenging property either directly via the redox potential of thiols, or secondarily via increasing glutathione levels in the cells [24]. These findings indicated that NAC may function as an effective suppressor of ROS production in PgLPS-treated cells.

It has been shown that autophagy induced by cellular stress is regulated in part by AMPK activation [25, 26]. We next performed western blotting to investigate whether intracellular production of ROS can regulate PgLPS-induced autophagy through AMPK activation (Fig. 6b). Phosphorylated AMPK (p-AMPK) expression was upregulated in pgLPS-treated keratinocytes, whereas its expression was suppressed in cells treated with NAC. In line with p-AMPK expression, treatment with NAC attenuated expression of beclin-1 and LC3-II, which was induced in PgLPS-stimulated cells. These findings suggested that intracellular accumulation of ROS may accelerate autophagy via the upregulation of AMPK activity.

### Toll-like receptor-4 (TLR-4) dependent signaling is associated with PgLPS-induced autophagy

**Fig. 7:**
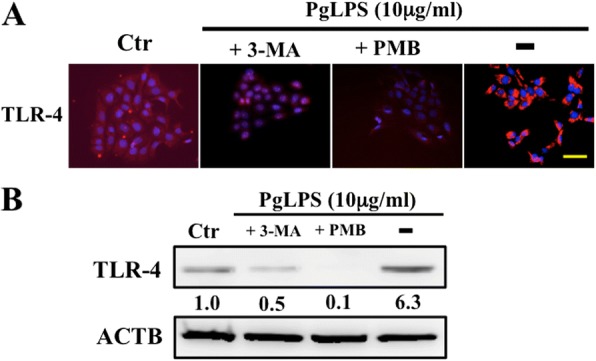
Induction of autophagy is dependent on TLR4 expression in PgLPS-stimulated HaCaT cells. HaCaT cells were stimulated for 24 hours in the absence (control, Ctr) or presence of PgLPS (10 μg/ml) with or without 3-MA (10 mM) or PMB (100 μg/ml). **a** Representative images of immunocytochemical expression of TLR-4 (Red) in HaCaT cells. Nuclei were stained with Hoechst 33342 (blue). Bar = 25 μm. **b** Western blotting analysis of TLR-4. β-actin (ACTB) was used as a loading control. Band densities were presened as antibodies’ expression fold-increases (normalized to ACTB) and compared with the results for untreated cells (Ctr). Quantification results are shown below the corresponding blots. All experiments were performed in quadruplicate

LPS is a ligand for TLR-4, and activates TLR-4 signaling to regulate multiple cellular effects [27]. We examined whether activation of TLR-4 signaling is associated with PgLPS-induced autophagy in keratinocytes using immunocytochemical and western blotting assays. Immunocytochemical results showed increased expression of TLR-4 in keratinocytes treated with PgLPS (Fig. 7a). Treatment with 3-MA and PMB decreased immunocytochemical expression of TLR-4 in PgLPS-stimulated keratinocytes. In western blotting, PgLPS-stimulated keratinocytes showed significantly increased expression of TLR-4 (Fig. 7b). Upregulation of TLR-4 expression was related to increased expression of LC3-II and beclin-1 (Fig. 5e). In contrast, 3-MA and PMB pretreatment decreased TLR-4 expression (Fig. 7b), indicating that inhibition of autophagy induction and LPS binding, respectively, can influence the expression of TLR-4 expression. These results demonstrated that acceleration of autophagy response by PgLPS was associated in part with TLR-4 signaling in HaCaT cells.

### PgLPS-induced autophagy enhanced co-localization of *E. coli* BioParticles with autophagosomes

**Fig. 8:**
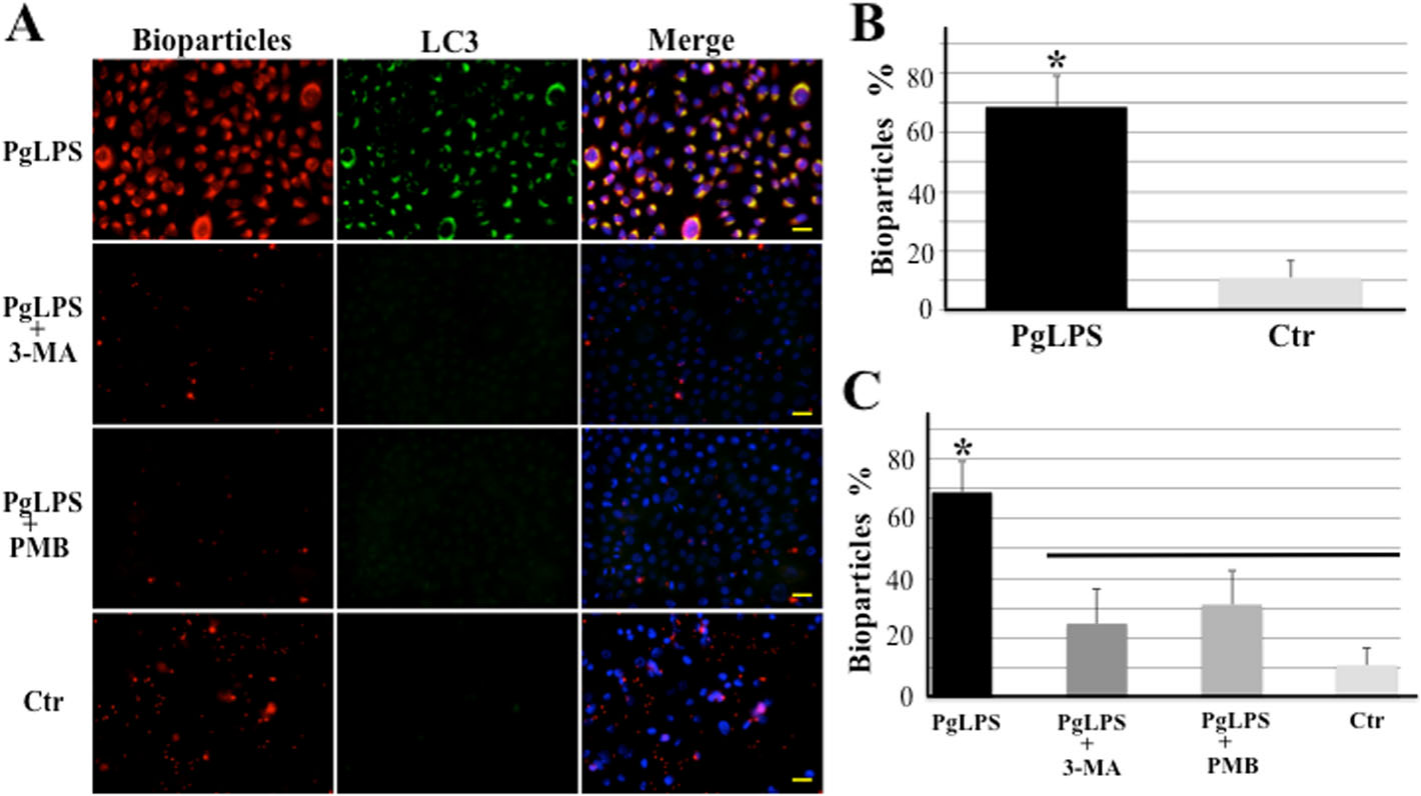
Co-localization of *E. coli* BioParticles with autophagosomes is promoted by PgLPS-induced autophagy. HaCaT cells were infected with Alexa Fluor 568-labeled *E. coli* BioParticles for 1 h. Following phagocytosis, HaCaT cells were treated for 24 h under control condition (Ctr), pgLPS (10 μg/ml), or pgLPS + 3-MA (10 mM), and pgLPS + PMB (100 μg/ml). **a** Representative fluorescence images of co-localization between bioparticles (red) and LC3-II-positive autophagosomes (green). Nuclei were stained with Hoechst 33342 (blue). Bar = 25 μm. **b** Quantification of the co-localization of bioparticles with LC3-II-positive autophagosomes in HaCaT cells treated with or without PgLPS. The graph shows the means ± SD from five independent studies. *Significantly different (Student’s t-test) at *P* < 0.05 compared with control (Ctr). **c** The graph shows quantification of co-localization of *E.coli* BioParticles with autophagosomes in HaCaT cells pretreated with or without inhibitors. All values are presented as the means ± SDs from five independent studies. *Significantly different at *P* < 0.05 compared with control (Ctr), PgLPS+ 3-MA, and PgLPS+PMB (ANOVA followed by Scheffe’s test). There are no significant differences between groups joined by horizontal bars

From our results using exfoliative specimens from keratinocytes (Figs. 1, 2, 3 and 4), we speculated that the cells appear to internalize bacteria in their environment exposed to bacterial LPS in the gingival sulcus. To examine whether PgLPS-induced autophagy causes recruitment of bacteria into LC-II-positive autophagosomes, we performed a phagocytosis assay with cultured keratinocytes using *E.coli* BioParticles. First, we immunocytochemically examined internalization and co-localization of bioparticles with autophagosomes in PgLPS-induced keratinocytes. Following treatment with PgLPS, cells were infected with fluorescent bioparticles and autophagosomes were stained with LC3-II. Immunocytochemical detection showed co-localization of *E. coli* BioParticles and LC-II-positive autophagosomes in pgLPS-induced keratinocytes (Fig. 8a). Control cells showed a few, scattered particles in extracellular spaces. Intracytoplasm of PgLPS-stimulated cells contained tiny aggregates of co-localization of particles and LC-II-positive autophagosomes. In PgLPS cells with 3-MA and PMB treatment, both aggregates of particles and LC-II-positive autophagosomes were abolished. As shown in Fig. 8b, the percentage of co-localization of bioparticles with LC-II-positive autophagosomes in PgLPS-treated cells was 68.8 ± 11.4%, while in control cells it was 10.8 ± 5.9% (*p* = 0.024). To further investigate the role of autophagy in the internalization of *E. coli* bioparticles, we examined co-localization of bioparticles with LC-3-II-positive autophagosomes in HaCaT cells treated with 3-MA and PMB. As expected, suppression of autophagy or TLR-4 signaling by 3-MA and PMB, respectively, attenuated co-localization of baioparticles with autophagosomes. The percentage of co-localization of particles with autophagosomes in Pg-LPS-stimulated cells was 68.8 ± 10.5%, while in 3-MA- or PMB-pretreated cells, it was 24.8 ± 11.4% (*p* = 0.036) or 31.2 ± 11.4% (*p* = 0.040), respectively (Fig. 8c).

## Discussion

Although bacterial autophagy system was recently found to target intracellular bacteria [28], an induction of autophagy in the sulcular epithelial keratinocytes remains unknown. In the present study we demonstrated that LPS could induce autophagy in both exfoliative sulcular and cultured keratinocytes. Exfoliative epithelial keratinocytes showed expression of LPS-induced proteins and co-localization of bacteria with autophagosomes. In cultured keratinocytes, PgLPS induced autophagy which was related to upregulation of AMPK activity through ROS and was in association with TLR-4-dependent signaling. Moreover, PgLPS enhanced internalization of bacteria-loaded bioparticles and promoted co-localization of the particles with autophagosomes. Treatment with 3-MA, a microtubule-disrupting agents, significantly attenuated internalization of bacterial-loaded bioparticles and their co-localization with autophagosomes induced by pgLPS treatment. These findings collectively suggest that interaction between bacteria and keratinocytes is mediated in part by LPS-induced autophagy.

In this study, an approach using exfoliative cytology showed interaction between bacteria and surface epithelium in the gingival sulcus. Internalization of bacteria, stained using both Giemsa and Papanicolau staining, was confirmed by intracytoplasmic expression of LBP. LBP is a glycoprotein synthesized predominantly by liver and is released into the bloodstream during an inflammatory response [29]. The normal plasma level of LBP can be upregulated dramatically after inflammatory stimulation such as sepsis induced by gram-negative bacteria. Specifically, LPS-initiated inflammatory response is significantly enhanced by LBP [29]. Because it is known that LBP binds LPS via recognition of lipid A [30], our immunocytochemical results of LBP staining are supported by recent report that has examined intracellular expression with LBP in human gingival tissues [31] and can be explained as an intracytoplasmic accumulation of bacteria-produced LPS in several sulcular keratinocytes.

Results of immunocytochemistry and western blotting using exfoliative cells presented here indicate that intracellular bacteria are harbored in autophagosomes of sulcular keratinocytes following LPS-induced autophagy. Stimulation of sulcular keratinocytes by bacterial LPS results in upregulated expression of ICAM-1, TLR4-, and TLR-2. TLRs initiate a series of innate immune mechanisms against various microorganism infections after sensing presence of LPS [27]. Intracellular expression of TLR-4 and TLR-2 was confirmed by recent report concerning immunohistochemical detection of them in pocket epithelial cells in periodontitis [32]. ICAM-1 expression is also induced by local stimulation of LPS, causing migration of neutrophils and T cells to surface epithelium [33]. These LPS-inducible factors may mediate an immunological reaction against periodontal diseases in the gingival sulcus. Furthermore, bacterial LPS also induced co-localization of bacteria and autophagosomes in sulcular epithelial cells. The ability of autophagy (i.e., xenophagy) to remove large cytoplasmic structures is used to clear intracellular bacteria, parasites, and viruses [10, 28]. Some human pathogens are degraded in vitro by xenophagy, including bacteria (e.g., group A streptococcus, Mycobacterium tuberculosis, and Salmonella enteric), viruses such as herpes simplex virus type 1 (HSV-1), and parasites such as Toxoplasma gondii [9]. Therefore, our results using exfoliative cytology suggest that sulcular epithelial cells, as the first line of host defense, may induce autophagy to trap bacteria, in addition to the innate immune response via TLRs.

The sulcular epithelium mainly consists of multilayered continuously renewing keratinocytes that necessarily contribute to defensive responses against LPS-producing periodontal bacteria in the gingival sulcus. LPS stimulation can induce autophagy in cultured keratinocytes [18, 34]. An increasing number of findings indicate that autophagy plays an important role in keratinocyte biology and pathology including keratinocyte-related mucocutaneous disorders, such as psoriasis and squamous cell carcinoma as well as periodontal diseases [21, 34]. This study demonstrated that cultured keratinocytes treated with PgLPS showed upregulated expression of LC3-II and beclin-1, indicating enhancement of autophagy. LC3-II and beclin-1 play important roles in autophagy in mammalian cells. In contrast to LC3-II, a marker of final autophagosome formation, beclin-1 participates in the early stages of autophagy, promoting the nucleation of the autophagy vesicle and recruiting proteins from the cytosol. During the process of autophagosome formation, the maturation stage is promoted by the interaction between LC3-II and beclin-1 [6, 9]. We observed that 3-MA or PMB-mediated blockage of autophagy or LPS-binding, respectively, attenuated autophagy in cultured keratinocytes treated with PgLPS. These results indicate that PgLPS is a general stimulant of beclin-dependent autophagy activity in human keratinocytes. In addition, our study showed that viability of PgLPS-treated cells was not significantly different from that of untreated cells. A recent study showed that exposure of cultured cells to LPS resulted first in autophagy and then, in apoptosis [35].

LPS-induced cellular stress results in increased cellular production of ROS [23, 36]. Intracellular ROS regulates multiple cellular functions such as DNA synthesis [37], transcription factor activation [37], gene expression [38], and proliferation [39]. Our results on intracellular ROS with or without NAC showed that PgLPS stimulation promoted intracellular ROS accumulation in cultured keratinocytes, suggesting that ROS acts as a cellular secondary messenger [37]. Increased intracellular ROS due to LPS accelerates activation of AMP-activated protein kinase (AMPK), because AMPK is an energy sensor activated by increase in ROS-induced cellular stress [40]. The enzyme activity of AMPK is dependent on phosphorylation of the PRKAA/a-subunit on Thr172 and can be conveniently monitored by western blotting with a phosphospecific antibody against this site [41]. Western blotting results showing increased pAMPK expression presented here imply that treatment with PgLPS accelerates activity of AMPK in cultured keratinocytes. Activated AMPK appears to stimulate autophagy in several cell types including fibroblasts, colon carcinoma cells, and skeletal muscle cells [42–44]. Recent studies revealed that activated AMPK was involved in processes that synergize to activate autophagy, by directly activating UNC-51-like autophagy activating kinase 1 (ULK1), a key initiator of the autophagic processes, and indirectly impairing mammalian target of rapamycin (mTOR)-dependent inhibition of ULK1 [44–46]. Furthermore, using pharmacological inhibition of ROS, we found that intracellular ROS can promote TLR-4 expression leading to enhancement of autophagy in cultured keratinocytes treated with PgLPS. It was reported that TLR-4 can mediated the cross-talk, between autophagy and immune signaling in keratinocytes [34]. These findings suggest that increased ROS accumulation due to PgLPS stimulation may preferentially promote autophagy via both AMPK and may be in association with TLR-4 pathways.

Bacterial autophagy has been increasingly recognized as an important defense mechanism to clear intracellular microbes, because bacterial pathogens are targeted by the autophagy pathway [28]. We therefore wondered whether induction of autophagy could affect internalization and recruitment to autophagosomes of *E. coli* bioparticles in HaCaT cells. We found that PgLPS-induced autophagy in infected HaCaT cells could lead to recruitment of particles within autophagosomes. Moreover, we observed that 3-MA or PMB-mediated blockage of autophagy or LPS-binding, respectively, suppressed co-localization of *E. coli* bioparticles with autophagosomes, leading to a loss of bioparticle uptake activity of cells. Taken together, these data demonstrated that the effect of PgLPS on bacterial internalization and uptake activity was dependent on the induction of bacterial autophagy.

We acknowledge a possible limitation in this study. This study may be limited by lack of direct evidence as to whether PgLPS-induced autophagy resulted in antibacterial effects. Although bacterial autophagy or xenophagy has been recognized as an important defense mechanism to clear intracellular microbes, recent studies postulated that some bacterial pathogens have evolved mechanisms to evade autophagic recognition or even co-opt autophagy machinery as a replicative niche for their own benefit [38, 47]. In this study, we collected exfoliative keratinocytes from normal-appearing gingival sulcus of periodontitis-free volunteers. The cells showed co-localization of bacteria with autophagosomes formed due to LPS-induced autophagy. These findings indicate that in the periodontitis-free gingival sulcus, autophagy induced by resident bacteria via their intake into autophagosomes, preventing stimulation of periodontitis. Therefore, we suggest that LPS-induced autophagy in sulcular keratinocytes may play a protective role in a maintenance of homeostasis in the periodontitis-free gingival sulcus. Further and more precise in vivo and in vitro studies may shed light on how PgLPS-induced autophagy combats invasive pathogens inside sulcular keratinocytes.

## Conclusion

The present study revealed that PgLPS-induced autophagy in either exfoliative sulcular or cultured keratinocytes accelerates co-localization of bacteria with autophagosomes. In cultured keratinocytes, PgLPS-induced autophagy was dependent partially on the activation of TLR4 signaling and the AMPK pathway via increased intracellular ROS. These results indicate that PgLPS-induced autophagy is at least partially responsible for the interaction between bacteria and sulcular keratinocytes in the gingival sulcus.
